# The social accountability of nursing training institutes in Morocco: The knowledge, perceptions and realization of its aspects

**DOI:** 10.30476/JAMP.2021.90618.1419

**Published:** 2021-10

**Authors:** MARIA BENIJJANE, MAJDA SEBBANI, LATIFA ADARMOUCH, OUASSIM MANSOURY, MOHAMED AMINE

**Affiliations:** 1 Community Medicine and Public Health Department, Research Laboratory, Biosciences and Health, School of Medicine, Cadi Ayyad University, Marrakech, Morocco; 2 Clinical Research Unit, Mohammed VI University Hospital, Marrakech, Morocco

**Keywords:** Social accountability, Nursing students, Knowledge, Medical school, COVID-19

## Abstract

**Introduction:**

The social accountability (SA) challenges health professional training institutes to reorient their missions to train the graduates aligned with the society's priority needs and produce quality, population centered care. The objective was to assess the knowledge, perceptions, and implementation of SA by nursing education institutions.

**Method:**

The cross-sectional observational survey was conducted at the Higher Institute of Health Professions and Techniques of Marrakech (ISPITS-M) and its annexes between May 17 and June 4, 2020, using a mixed methodology. The data were collected through an online questionnaire, which was tested with 11 students and teachers. It was completed by 50 teachers and 213 students, recruited on a voluntary basis and was developed based on the literature review, aspects and principles of social accountability. In addition, eight semi-structured interviews were performed with administrative staff recruited by purposive sampling.

**Results:**

Out of a total of 924 students and 75 teachers, 213 students and 50 teachers participated in the survey, with a response rate of 23% and 67% simultaneously. The average age was 40.2±8.9 years for the teachers and 19.7±1.3 years for the students. 36% of the students had no knowledge of SA; teachers and leaders had different perceptions and the concreteness of the aspects of SA was low. The students felt they were less prepared in social determinants of health (85%), community lifestyles to be served (85%), outreach care, and frontline work (83%).

**Conclusion:**

The knowledge of SA is average; there is a need to raise awareness of SA in addition to strengthening training programs and the concretization of actions in this direction.

## Introduction

In December 2019, a new disease, COVID -19, emerged in Wuhan, China. In January 2020, the outbreak was declared by the World Health Organization (WHO) as a public health emergency of international concern ( 1 , 2 ). The COVID-19 pandemic represented a surprising event that caused changes in our training institutes and prompted the application of rapid management and adaptation measures requiring the accompaniment and support of teachers, students and administrative staff ( 3 ), adoption of specific skills and roles towards the community as a response to the mandate of social accountability (SA).

 SA must be concretized in each level of health professional training, in the institute's activities, such as a thorough knowledge of the population served by the institution, and research and teaching methods in order to have the capacity to anticipate health problems such as those during the COVID-19 health crisis and thus align training, research and service activities ( 4 - 8 ).

 In Morocco, following the first case of COVID-19, declared on March 02, 2020, the country has undertaken a preventive approach to rapidly address the pandemic. Since March 16, 2020, the country has announced the suspension of classes in all educational, vocational training, and university institutions, as well as the suspension of internships, particularly for health training institutes ( 4 - 8 ). These measures require the mobilization and adhesion of all socio-professional and institutional sectors, including the training institutes for health professionals as actors in the health systems by demonstrating their SA ( 9 , 10 ).

 The SA of health professional training institutions was defined by WHO in 1995 as the obligation to direct education, research and service activities towards solving the priority health problems of the community or nation they are mandated to serve. Priority health concerns must be jointly identified by governments, health care organizations, health professionals, and the public ( 11 , 12 ).

 Thus, SA is the practice of engaging with communities that the health training institute is mandated to serve, anticipating and responding to their current and future priority health needs and societal challenges, creating and strengthening governance and partnerships with other stakeholders, and demonstrating and verifying the impact of their actions on the health of society ( 13 ). 

 In this context, it is important to appreciate the concretization of SA by our health training institutes through the aspects reflecting it, to identify the perceptions and knowledge of the professionals and students of these institutions of the concept and their SA towards the community in the context of the pandemic COVID-19. The present study concerned the Higher Institute of Nursing and Technical Health Professions of Marrakech (ISPITS-M) and its region (Essaouira and Safi). 

## Methods

### Type of study and context

 This was a cross-sectional observational study of the Higher Institute of Health Professions and Techniques of Marrakech (ISPITS –M) and its region, which was conducted between May 17 and June 4, 2020, following a mixed methodology. ISPITS-M is a higher education institution that does not fall under the jurisdiction of universities and is under the supervision of the governmental authority in charge of health. Its main missions are basic and continuing education, scientific and technological research, renewal of nursing and health technology practices, organization of nursing services. The studies are organized in three cycles (professional license, master’s, and doctorate). 

### Recruitment of participants

 The participants in the quantitative study were undergraduate nursing students of all levels and options in addition to permanent and temporary teachers of the institute, taking into account a non-probability sampling, participation was voluntary and the answers were de-identified. As for the qualitative part, the interview participants were selected by purposive sampling. The inclusion criterion was to be a manager or leader (director of an institute, director of studies, head of a student affairs unit, etc.) and the exclusion criterion was refusal to participate in the study. Participants were invited via their email address as well as social networks WhatsApp, Facebook.

### Data Collection

 The quantitative data were collected using a self-administered electronic questionnaire, developed based on the literature review, aspects and principles of social accountability. It was tested with 11 participants who were then excluded from the study sample. It included 3 types of questions:

- Binary (yes or no) or multiple-choice questions.
- A Likert scale with levels
- Open-ended questions

The questionnaire was designed in 4 parts: Socio-demographic characteristics of the participants, Aspects concretizing AS, Knowledge of the concept of AS, and Students' perceptions of their AS. The data were extracted by Excel and then analyzed by SPSS version 21. Descriptive and bivariate analyses were performed. The chi-square test was used to compare the percentages (significance level was 0.05).

 The qualitative data were collected through eight semi-structured interviews conducted with administrative staff using an interview guide and recruited through purposive sampling. The themes were related to the perception, definition and knowledge of SA, and the management of the COVID-19 crisis by the Institute. They were transcribed verbatim; then, the themes were coded, the relevant sentences highlighted, and the answers analyzed by the content analysis method. 

### Regulatory and ethical considerations

Confidentiality and anonymity rules for analysis and data collection were followed, and the participants’ consent was obtained.

### Ethics committee opinion

This is an opinion survey and according to the Moroccan biomedical law, the approval of the ethics committee was not requested.

## Results

### Description of the participants

 The number of participants who responded to the questionnaire was 263; of them, 50 were teachers and 213 were students, with a response rate of 23% among students and 67% among teachers (N: 924 students and 75 teachers). Among the teachers, 58% were male, while for the students, the majority were female (80%). The mean age was 40.2±8.9 years for the teachers and 19.7 ±1.3 years for the students (Table 1). 

**Table 1:** Socio-demographic characteristics of the participants

	Variables	Students	
		Number(%)	Number(%)
Gender	Male	29(58.0)	42(19.8)
	Female	21(42.0)	171(80.2)
Marital status	Single	9(18.0)	207(97.1)
	Married	40(80.0)	6(02.9)
	Divorced	1(2.0)	00(0.0)
Profile	Permanent	32(60.0)	
	Temporary	18(40.0)	
Year of study	1st		95(44.6)
	2nd		68(32.0)
	3rd		50(23.4)

### Aspects of ISPITS-M SA during the COVID-19 pandemic

 The majority of the participants argued that the disruption caused by the COVID-19 pandemic and the actions taken by ISPITS to adapt and manage it, in this case the adoption of distance learning, required the commitment of resources, including platforms, adequate preparation and the appropriation of specific skills. As for the preparedness of the institutions to manage the COVID-19 pandemic or other health crises in the future, 58% of the teachers stated that the level of preparedness was average. In addition, the majority of the students felt that they were less prepared, especially in the areas of social determinants of health (85%), lifestyles of the community to be served (85%), outreach and frontline work (83%), prevention techniques (54%), communication (58%); and collaborative work (61%). Thus, the administrative staff emphasized that they were never prepared for such a situation: “it must be said that we were not prepared”. The participants pointed out that the resources devoted by the institute to produce pedagogical content were weak; according to them, “it is necessary to reinforce the computer equipment”, “it is necessary to establish a worthy E-Learning platform”.

 Also, 65% of the students pointed out that the incentive made by the institute for them to acquire the necessary skills to face this pandemic or other health crises in the future was insufficient and average, especially those related to information and communication technologies (ICT). Also, the administrative staff reported that the adoption of these skills was average or even weak for most of them; they stated, “we didn't know what distance learning is”, “at the beginning, it was difficult for teachers to adhere to these solutions”, and “it was more of a training problem”.

 Regarding the accompaniment and support of students and teachers by the institute during the COVID-19 pandemic, only 22% of students and 50% of teachers expressed satisfaction. There was no statistical difference between teachers and students (P<0.001), respectively. The managers expressed the lack of support or accompaniment from the leaders and that they were alone while trying to work with the means available: “... the problem we had was that we were all alone” and “we had to innovate everything”. 34% of the teachers and 20% of the students expressed that the anticipation and response to their expectations was unsatisfactory (P= 0.001). 42% of the teachers carried out individual actions towards the community during this pandemic, in relation to raising awareness of the population on the prevention measures of COVID-19 and involvement in distance learning, compared to 58% who did not carry out any actions. Among the teachers, 80% affirmed that the institute was committed to the community that it had the mandate to serve, especially through the training of health professionals (85%), little through research (6%) and services (9%) (Table 2).

**Table 2:** Aspects concretizing the SA of ISPITS-M

Variables	Teachers		Students		P*
	Number(n)	Percent(%)	Number(n)	Percent(%)	
Level of satisfaction with the level of guidance and support for nursing students and teachers by the institute during the COVID-19 pandemic.					
Very dissatisfied	1	2.0	13	6.1	<0.001
Insatisfied	10	20.0	41	19.2	
Neutral	12	24.0	109	51.1	
Satisfied	26	52.0	47	22.0	
Very satisfied	1	2.0	03	01.4	
Institute's level of preparedness for pandemic management					
Low	4	8.0			
Medium	29	58.0			
Well	17	34.0			
Incentives for students by the institute to acquire the necessary skills to deal with the pandemic					
No			31	14.5	
Medium			140	65.5	
Well			42	20.0	
Anticipating and responding to student expectations during the pandemic period					
Unsatisfactory	10	20.0	73	34.2	0.001
Neutral	15	30.0	90	42.2	
Satisfactory	25	50.0	50	23.4	
Implementation of actions towards the community during the pandemic period					
Yes	21	42.0			
No	29	58.0			
Participation of students and teachers in the planning of actions affecting teaching/learning implemented by the institution					
Yes	25	50.0	59	27.7	0.003
No	25	50.0	154	72.3	

* P=Statistically highly significant,

* SA: Social Accountability,

* ISPITS-M: Institute of Health Professions and Techniques of Marrakech

### Knowledge of the concept of SA of health training institutions and perceptions of SA during the pandemic COVID-19

 The knowledge of SA was average: 36% of students had no knowledge of the concept of SA. As for students' social accountability, 45% considered that their SA lay in complying with health measures, focusing on their studies in 11% of cases and 2% answered "no" and that students did not really have a responsibility to society (Table 3).

**Table 3:** Student nurses' knowledge and perceptions of their SA during the pandemic

Variables	Students	
	Number(n)	Percent(%)
Students' knowledge of the concept of social accountability		
Yes	136	64.0
No	77	36.1
Students' perceptions of their SA during the COVID-19 pandemic		
No accountability	5	2.3
Study	24	11.2
Inform, Educate	70	33.0
Participate in health actions	10	5.0
Respect of sanitary measures	97	45.5
Social solidarity	7	3.2

* SA: Social Accountability

 Based on the results, the knowledge of SA was average; 36% of the students had no knowledge of the concept; also, teachers and leaders had different perceptions, even unfamiliarity with the concept, as was illustrated by their words: “it is a concept I have never heard in training institutes"; "our SA is firstly our mission to solve the students' problems"; "...no idea"; ". ...To help and be responsible"; "… is to assume our basic mission and to continue the teachings"; and "this is the institutional role of ISPITS as a public and civic institution of training, research and development to reduce the effects of the pandemic”…. In addition, 46% of the students stated that their SA during this pandemic was to comply with health measures (Table 3).

## Discussion

 Thus, SA is a guideline and value that must be adopted by all actors in the institute; its aspects must be reflected throughout the training process and maintained after recruitment, from the conceptualization of the product (student) to its use, in all contexts and settings, namely the context of health crises such as the COVID-19 pandemic ( 14 - 21 ). 

 The COVID-19 has affected the activities of health training institutes, which are obliged to adapt to it and participate in the management of the pandemic through initiatives that respond to their SA. This study examined the realization of the SA by ISPITS-M by reflecting its aspects, as well as the perceptions of professionals and students of the concept and their SA towards the community in the period of the pandemic COVID-19. 

 The study revealed that COVID-19 has impacted the activities of ISPITS-M, especially in the education component. The institute has adopted distance learning which requires the adaptation of the educational process to meet the needs and expectations of students in these different conditions. However, the teachers and students’ satisfaction with the methods adopted and their preparation was average, especially the appropriate skills. Ideally, the selection of methods and alternatives that meet the needs of students in the changing conditions is a guarantor of quality teaching as an aspect of SA ( 16 ). However, this will affect the quality of the training and improvement of the performance of professionals as well as the adaptation of skills to the rapidly changing context and local conditions ( 15 , 22 ).

 Both faculty and leadership asserted the poor resources available and committed by the institute, including the lack of an online training platform of the institute. Lindgren and Karle ( 16 ) point out that the SA of health training institutions must be demonstrated in several aspects, namely the availability of adequate facilities, clinical platforms that will support the students' learning and, hence, the importance of unlimited access to ICT ( 18 ).

 Most participants expressed that they were less prepared to deal with the pandemic, especially in terms of the social determinants of health, knowledge of society and its changing lifestyles, and health promotion. The SA challenges the health training institutions to address these determinants through teaching about them, undertaking health promotion and primary care interventions, and caring for vulnerable groups, including knowing the community and anticipating and responding to their current and future health problems ( 21 , 23 ).

 The staff and leaders of ISPITS-M stated that their institute had not organized actions towards the society, except for some individual initiatives led by teachers and students through public awareness; however, the SA encourages the institutes to improve the performance of professionals through interventions that promote the health of all the clients ( 24 ). Also, according to Wang et al. ( 1 ), the contribution of universities in the control and risk management of epidemics is a function and responsibility of universities.

 However, the data explored in this study highlighted the lack of support and guidance for students, teachers, and administrators in this period of crisis and the emergence of new educational, social and health needs that will affect their motivation and commitment to training and to the community. This corroborates with Mukhtar et al.’s findings ( 18 ); they reported that emotional and social support was involved in improving the students’ motivation and was essential for effective learning.

 The study revealed an average level of knowledge of the concept of SA. In addition, the participants expressed different perceptions of their SA, specifically in this period of pandemic; the majority of them demonstrated a deficit in the knowledge of SA of health training institutes. The same finding was reported by Ritz et al. ( 25 ) regarding the understanding of the concept of SA by training institutes, which remains ambiguous and limited. According to Preston et al. ( 26 ), all stakeholders need to have an explicit, universal, and common understanding of SA including students, teachers, institute leaders, and community in order to facilitate the adoption of SA and buy-in to the SA movement. Participation in the prevention and control of disease, including the organization of support and care for the vulnerable groups and promotion of health to improve their health and living conditions, is a sign of a socially accountable educational institution.

## Advantages and limitations

 Since its emergence, the concept of SA has aroused a growing interest in medical schools, trying to promote and develop it, particularly through scientific research that is more focused on medical training. In Morocco, our study is the first one conducted in training institute for the nurses, and there was an attempt to explore the knowledge and perception as well as the concretization of SA, who are part of the actors of the care system. The results showed that the concept of SA in health training institutes was still poorly known by nursing students, teachers and even ISPTIS_M directors, and that it is ambiguous and requires more investigation, research and information and training actions on SA and its practice. In this context, a research study is still being carried out among the training institutes of health professionals in Morocco within the framework of a research work of doctorates in science fields.

 The limitations of the study were the low response rate among students, as well as the information bias related to the declarative aspect of the answers, which must be taken into consideration.

## Conclusion

 The SA is prompting the training institutes to reorient their training, research, and service activities to produce professionals aligned with the health priorities of the community to be served, with sufficient skills to work effectively and meet the current and future challenges of the society. ISPITS-M, through its students, faculty, and leadership, seemed to be moderately prepared to embody and reflect aspects of SA during the pandemic. It would be important to seize the post-crisis period to raise awareness about SA and concretize more actions in this sense.

## References

[1] Wang C, Cheng Z, Yue XG, McAleer M ( 2020). Risk Management of COVID-19 by Universities in China. J Risk Financ Manag.

[2] Zhou T, Huang S, Cheng J, Xiao Y ( 2020). The Distance Teaching Practice of Combined Mode of Massive Open Online Course Micro-Video for Interns in Emergency Department During the COVID-19 Epidemic Period. Telemed E-Health.

[3] Araújo FJ de O, de Lima LSA, Cidade PIM, Nobre CB, Neto MLR ( 2020). Impact Of Sars-Cov-2 And Its Reverberation In Global Higher Education And Mental Health. Psychiatry Res.

[4] Nani S Le Marco Face Au Covid-19. Rev Marocaine Santé Publique [Internet]. 18 Mai 2020 [cité 18 oct 2020];. https://revues.imist.ma/index.php/RMSP/article/view/20959.

[5] El Kahkahi  R, Moustaine M, Hafidi M, Zouhair R, Errakhi R (2020). Coronavirus disease (COVID-19) in Morocco: situation update and proposed remedial measures. Germs.

[6] Draissi Z, ZhanYong Q COVID-19 Outbreak Response Plan: Implementing Distance Education in Moroccan Universities. SSRN Electron J [Internet]. 2020 [cité 16 oct 2020]. https://www.ssrn.com/abstract=3586783.

[7] Hantem A, Saili S, Mssatfa H, Soubai H, Elmellouki M, Lazaar L, et al ( 2020). Les conditions de l’enseignement à distance pendant le confinement dû au COVID19: Cas de l’enseignement supérieur au Maroc. Arch-Ouvert.

[8] El Bettioui  R ( 2020). Usage et utilité des plateformes de la formation digitale des enseignants pendant la crise de Covid-19. Revue Française d’Economie et de Gestion.

[9] Boelen C, Heck J (1995). Defining and measuring the social accountability of medical schools.

[10] Grand’Maison P, Ladner J, Maherzi A, Poitevien G, Poitras J, Duplain R, et al ( 2015). Facultés de médecine francophones et responsabilité sociale: approche stratégique 2015-2020. Pédagogie Médicale.

[11] Boelen C, Pearson D, Kaufman A, Rourke J, Woollard R, Marsh DC, et al ( 2018). Rendre une faculté de médecine socialement responsable: Guide AMEE n ^o^ 109. Pédagogie Médicale.

[12] Armstrong SJ, Rispel LC ( 2015). Social accountability and nursing education in South Africa. Glob Health Action.

[13] Boelen C, Pearson D, Kaufman A, Rourke J, Woollard R, Marsh DC, et al ( 2016). Producing a socially accountable medical school: AMEE Guide No. 109. Med Teach.

[14] Jamieson J, Snadden D, Dobson S, Frost H, Voyer S ( 2011). Health Disparities, Social Accountability and Postgraduate Medical Education. Memb FMEC PG Consort.

[15] Frenk J, Chen L, Bhutta ZA, Cohen J, Crisp N, Evans T, et al (2010). Health professionals for a new century: transforming education to strengthen health systems in an interdependent world. The Lancet.

[16] Lindgren S, Karle H (2011). Social accountability of medical education: Aspects on global accreditation. Med Teach.

[17] Cappon P, McMurtry R, Parboosingh J, Cruess R, Cruess S, Hawkins D, et al (2001). Social accountability: a vision for Canadian medical schools.

[18] Mukhtar K, Javed K, Arooj M, Sethi A Advantages, Limitations and Recommendations for online learning during COVID-19 pandemic era. Pak J Med Sci [Internet]. 18 Mai 2020 [cité 17 Oct 2020];36(COVID19-S4);. http://pjms.org.pk/index.php/pjms/article/view/2785.

[19] Congiusta DV, Otero K, Ippolito J, Thomson J, Beebe KS ( 2020). A new role for orthopaedic surgeons: ongoing changes, lessons learned, and perspectives from a level I trauma center during the COVID-19 pandemic. J Shoulder Elbow Surg.

[20] Elsanousi S, Elsanousi M, Khalafallah O, Habour A Assessment of the social accountability of the faculty of medicine at University of Gezira, Sudan [Internet]. 2016 [cité 16 oct 2020];. http://repo.uofg.edu.sd/handle/123456789/753.

[21] Woollard B, Boelen C ( 2012). Seeking impact of medical schools on health: meeting the challenges of social accountability, Seeking impact of medical schools on health. Med Educ.

[22] Pather N, Blyth P, Chapman JA, Dayal MR, Flack NAMS, Fogg QA, et al ( 2020). Forced Disruption of Anatomy Education in Australia and New Zealand: An Acute Response to the Covid-19 Pandemic. Anat Sci Educ.

[23] Ventres W, Boelen C, Haq C ( 2018). Time for action: key considerations for implementing social accountability in the education of health professionals. Adv Health Sci Educ.

[24] Buchman S, Woollard R, Meili R, Goel R ( 2016). Practising social accountability: From theory to action. Can Fam Physician.

[25] Ritz SA, Beatty K, Ellaway RH ( 2014). Accounting for social accountability: Developing critiques of social accountability within medical education. Educ Health.

[26] Preston R, Larkins S, Taylor J, Judd J ( 2016). Building blocks for social accountability: a conceptual framework to guide medical schools. BMC Med Educ.

